# Functional Assays for Measuring the Catalytic Activity of Ribosome Inactivating Proteins

**DOI:** 10.3390/toxins10060240

**Published:** 2018-06-14

**Authors:** Yijun Zhou, Xiao-Ping Li, Jennifer N. Kahn, Nilgun E. Tumer

**Affiliations:** Department of Plant Biology, School of Environmental and Biological Sciences, Rutgers University, New Brunswick, NJ 08901-8520, USA; yz334@gsbs.rutgers.edu (Y.Z.); xpli@sebs.rutgers.edu (X.-P.L.); jennifer.nielsen.kahn@gmail.com (J.N.K.)

**Keywords:** ribosome inactivating protein, depurination assays, translation inhibition assays

## Abstract

Ribosome-inactivating proteins (RIPs) are potent toxins that inactivate ribosomes by catalytically removing a specific adenine from the α-sarcin/ricin loop (SRL) of the large rRNA. Direct assays for measuring depurination activity and indirect assays for measuring the resulting translation inhibition have been employed to determine the enzyme activity of RIPs. Rapid and sensitive methods to measure the depurination activity of RIPs are critical for assessing their reaction mechanism, enzymatic properties, interaction with ribosomal proteins, ribotoxic stress signaling, in the search for inhibitors and in the detection and diagnosis of enteric infections. Here, we review the major assays developed for measuring the catalytic activity of RIPs, discuss their advantages and disadvantages and explain how they are used in understanding the catalytic mechanism, ribosome specificity, and dynamic enzymatic features of RIPs.

## 1. Introduction

Ricin, a plant toxin produced by *Ricinus communis* and Shiga toxins (Stx) produced by foodborne pathogens, *Shigella dysenteriae type 1* and Shiga toxin (Stx)–producing *Escherichia coli* (STEC) are ribosome inactivating proteins (RIPs) [1,2,3,4]. Ricin, STEC and *Shigella* are classified as category B agents of national security and public health risk with potential for significant morbidity and mortality [5]. STEC infection can lead to hemorrhagic colitis (HC) or hemolytic uremic syndrome (HUS), which is the most common cause of renal failure in infants and young children in the US [6]. Presently there are no FDA approved vaccines or therapeutics against ricin or Stx-producing bacteria. Ricin and Stxs are type II RIPs that contain a toxic A chain and a lectin B chain connected through a disulfide bond. Type I RIPs such as pokeweed antiviral protein (PAP), gelonin, saporin, and trichosanthin (TCS) contain only one chain, which corresponds to the A chain of type II RIPs. Type III RIPs such as maize RIP are also single chain but are synthesized as precursors and require removal of an internal peptide to become active [7]. The lectin B chain of the type II RIPs binds to glycoproteins or glycolipids to promote endocytosis of the toxin leading to higher toxicity of type II RIPs compared to type I RIPs [8].

Ricin is the earliest characterized and most widely studied RIP. The mechanism of action of ricin toxin A chain (RTA) was elucidated in 1987 by Endo et al., who showed that ricin cleaves the *N*-glycosidic bond at A_4324_ in the sarcin/ricin loop (SRL) of the 28S rRNA in rat liver ribosomes [9]. The SRL is part of the GTPase Associated Center (GAC) of the ribosome critical for binding of translation factors [10]. Depurination of the conserved adenine leads to inhibition of GTP hydrolysis, inhibition of protein synthesis and eventually apoptosis. Other RIPs, such as abrin, modeccin, Stx, PAP and trichosanthin (TCS) were shown to depurinate the same adenine in rat liver ribosomes [11,12,13]. RIPs irreversibly modify the SRL through their RNA *N*-glycosidase activity (EC 3.2.2.22), while ribotoxins such as α-sarcin also target the SRL, but cleave the SRL through their rRNA endonuclease activity (EC 3.1.27.10) [14,15]. RTA does not inactivate prokaryotic ribosomes although the SRL is conserved on the 23S and 28S rRNAs [16]. However, RTA could cleave the *N*-glycosidic bond at A_2600_ in the naked 23S rRNA as well as at other sites [16]. In contrast to RTA, PAP could depurinate A_2660_ in *E. coli* ribosomes [17] and A_4324_ in eukaryotic ribosomes [18]. These results suggested that ribosomal proteins play an important role in the ribosome specificity of RIPs [19]. Subsequent results indicated that ricin, Stx1, Stx2, and TCS interact with P-proteins of the ribosomal stalk to depurinate the SRL [20,21,22,23], while PAP binds to ribosomal protein L3 [24,25]. Interaction of RTA with P-proteins, which differ between eukaryotic and prokaryotic ribosomes, was suggested to be responsible for its specificity for eukaryotic ribosomes [20]. The interaction site of P-proteins with a single chain RIP, trichosanthin (TCS) was mapped to a conserved 11-mer peptide, SDDDMGFGLFD (P11) present at the *C*-terminal domain (CTD) of all P proteins [26]. This interaction is primarily mediated by the electrostatic interactions of K173, R174, and K177 in the *C*-terminal domain of TCS with the conserved DDD residues in the CTD of P-proteins along with hydrophobic interactions, which play a vital role in the stabilization of bilateral contacts between TCS and P11 [23]. The recent crystal structure of RTA with P11 showed that the last six residues of the stalk proteins (GFGLFD) docks into a hydrophobic pocket at the *C*-terminus of RTA [27,28]. The negatively charged (SDDDM) motif at the amino terminal end of P11 was not defined in the crystal structures [27,28]. The structural superposition of the TCS-P2 and RTA-P2 complexes demonstrated that the P2 peptide adopted distinct orientations and different interaction modes with the two different RIPs, suggesting the flexibility of the CTD in facilitating the access of RIPs to the ribosome [27,28]. The differences in ribosome specificity and in ribosome interactions of RIPs highlight the importance of assays that can distinguish between the activity of RIPs on the ribosome and on the naked rRNA and assays that can measure the relative activity of RIPs on ribosomes from different organisms.

RIPs are therapeutic agents and potential threats to public health and public safety. Assays that directly measure depurination activity for toxin detection and mitigation procedures were recently reviewed [29,30]. Here, we specifically review the assays, which identified differences in the activity of Stxs [31], differences in ribosome interactions of RTA [22,32,33,34,35,36], Stxs [21,22,37], TCS [23,26], and PAP [24,25], provided information about the downstream consequences of depurination, such as the ribotoxic stress response and apoptosis [38,39,40], allowed the use of RIPs as tools to probe the structure and function of the ribosomal stalk [41], provided information about the functional divergence of the ribosomal stalk P1-P2 dimers [42] and were useful in the search for RIP inhibitors [43,44,45,46,47,48]. We discuss the advantages and limitations of each method and explain how more sensitive assays can distinguish the differences in depurination activity and provide information about the kinetics and the mechanism of the depurination reaction.

## 2. Depurination Assays

### 2.1. Aniline Cleavage Assay

Aniline cleavage assay developed in 1987 was the first method to detect depurination activity of RIPs [11,49]. Endo et al., [49] used it to identify the site of depurination of RIPs. They showed that the mobility of 28S rRNA is altered after treatment with RTA and that RTA treated 28S rRNA became resistant to ribonuclease action. Subsequent evidence showed that RTA cleaves the *N*-glycosidic bond of A_4324_ in a hydrolytic fashion [9]. After removal of A_4324_, the 5′ and 3′ phosphodiester bonds of A_4324_ became unstable. Treatment with aniline at acidic pH caused cleavage of the RNA backbone at A_4324_ [49]. Aniline treatment released an RNA fragment (α-fragment) of about 400 nucleotides upon separation on a polyacrylamide gel [49]. The cleaved rRNA fragment is normally visualized by staining on a polyacrylamide gel.

### 2.2. Primer Extension Assay

This assay was used first by Iordanov et al. [38] in 1997 to monitor RTA induced depurination of A_4323_ in mammalian cells and was used to show that damage to 28S rRNA plays a critical role in initiating a signal transduction pathway called the ribotoxic stress [38]. The primer extension method uses a γ-^32^P-labeled primer, which anneals to the rRNA downstream of the depurination site. Depurinated rRNA isolated from RIP treated cells is incubated with γ-^32^P-labeled primer for amplification. Extension of this primer by reverse transcriptase using the depurinated rRNA as a template yields a product with a specific length due to stopped extension at the depurination site. The amplified product is separated on a 6% polyacrylamide gel containing 7M urea and visualized using a PhosphorImager. Using this method it was shown that RTA depurinates A_4324_ of the SRL in mammalian cells [38]. In subsequent studies, Parikh et al., developed a dual oligo primer extension assay to quantify the extent of rRNA depurination [50]. This assay involved labeling equimolar amounts of two oligonucleotides and hybridizing them to total RNA. One primer hybridized downstream of the depurination site and was used to examine the extent of depurination, while the other primer hybridized upstream of the depurination site and was used to quantify the total amount of rRNA [50]. The ratio of the depurination fragment compared with the control fragment allowed for accurate quantification of the extent of depurination. This dual extension assay was used to examine depurination by RIPs in vivo in yeast [20,22,34,50,51,52] and in mammalian cells [39,53,54].

### 2.3. qRT-PCR Assay

Melchior et al. [55] developed a quantitative real-time PCR method in 2010 that measured directly the enzymatic activity of RIPs on RNA. It took advantage of reverse transcriptase to insert an adenosine into the nascent complementary DNA (cDNA) at the abasic site where the template rRNA was depurinated, leading to T to A transversion at that site. The RNA was treated with the RIP first then reverse transcribed into cDNA. Quantitative real-time PCR (qRT-PCR) was carried out with two primer sets, one to detect total 28S rRNA and another one to detect the altered sequence [55]. This assay was designed to detect low levels of ricin in environmental samples and in food and thus it was conducted at 55 °C and the pH was lowered to 5.0 to detect RIP activity on RNA substrates [55].

In 2011, the qRT-PCR assay was adapted for detection of the catalytic activity of RIPs under physiological conditions using ribosomes as substrates [56]. The comparative cycle threshold (CT) method (ΔΔCT) with two primer pairs, one amplifying the target of interest and a second amplifying the total amount of input 28S rRNA as a reference amplicon, was used to quantitate the extent of depurination in vivo. The target reactions were normalized to the reference amplicon to measure the amount of depurinated RNA relative to the total amount of RNA. The base change at the depurination site was detected using a reverse primer with adenine at the 3′ terminus and the specificity for detection was enhanced by the inclusion of a secondary mismatch two base pairs 5′ to the adenine. The modified qRT-PCR assay provided a sensitive and highly reproducible measure of the extent of depurination by ricin and Stxs in mammalian cells [31,32,37,40] and in yeast [31,34,36,37] in vivo.

### 2.4. Adenine Detection Assay Using HPLC

High-pressure liquid chromatography (HPLC) was the first method adopted in 1989 to directly quantify released adenine from ribosomes with high sensitivity [57]. The method was based on the conversion of adenine into 1,N^6^-etheno derivative by chloroacetaldehyde and detection of adenine by HPLC with a fluorescent detector [57]. Zamboni et al. was able to use it to quantify released adenine from ricin or gelonin treated eukaryotic ribosomes [57]. Ribosomes were incubated with the RIPs followed by cold ethanol extraction. The ethanol-soluble fraction, which contained adenine in its etheno derivative form, became highly fluorescent after treatment with chloroacetaldehyde and this derivation step greatly enhanced the detection of adenine [57]. The chloroacetaldehyde derivative had to be further extracted with water-saturated diethyl ether and passed through a 0.45 µm filter [57] before it was applied to HPLC [57]. Using this method the protective effect of adenine against ricin was discovered [57], several RIPs were shown to depurinate different DNA, RNA, and poly(A) substrates [58,59] and in vitro conditions necessary for depurination of eukaryotic ribosomes were characterized [60]. The HPLC assay was used to examine the mechanism and kinetics of depurination of stem-loop RNA substrates by RTA and showed that the catalytic rate of RTA on RNA substrates was greatest at low pH (pH 4.0) and not at physiological pH [61]. Since RTA depurinates ribosomes at the physiological pH, these results provided evidence that the ribosomal proteins play an important role in depurination of the SRL by RTA [61]. To bypass the labor-intensive adenine derivatization step, mass spectrometry (MS) was introduced for the detection of adenine [62,63]. The HPLC-MS method was used to calculate RTA steady state kinetic parameters [62,63] and could be used to measure ricin activity in environmental samples [63].

### 2.5. Enzymatically-Coupled Adenine Detection Assays

Enzymatically-coupled assays were methods developed later to indirectly quantify released adenine and did not require adenine derivatization. In 2002, Heisler et al. described a colorimetric adenine quantification assay [64]. Adenine was converted into adenosine monophosphate by adenine phosphoribosyl transferase (APRTase) and the inorganic phosphates generated from adenine-AMP conversion reactions were quantified by a colorimetric method with comparable sensitivity to HPLC [64]. However, this method could not measure ribosome depurination in a continuous manner because phosphate and adenine interfered and required removal by dialysis before the assay. Despite this drawback, it was used to measure time and concentration dependent release of adenine by various RIPs [64] and to calculate kinetic constants.

In 2009, Sturm et al. developed an enzymatically-coupled assay with sufficient sensitivity to continuously measure adenine release from eukaryotic ribosomes [65]. It also relied on APRTase to convert adenine into AMP, but incorporated conversion of AMP into ATP by pyruvate orthophosphate dikinase (PPDK) to increase the sensitivity of detection. The ATP generated was quantified by firefly luciferase and the resulting AMP was rapidly converted to ATP by PPDK [65]. The enzymatically coupled luciferase assay had sufficient sensitivity to continuously measure adenine release from nanomolar concentrations of intact eukaryotic ribosomes and could detect femtomoles of ricin in minutes [65]. Continuous measurements could be carried out in a 96-well format and subpicomole levels of adenine could be quantitated in a continuous high throughput format. Since APRTase and PPDK used in the coupling reaction have optimal pH at 7, which is the same pH required for depurination of the ribosome by RTA, ribosome depurination could be measured continuously by this assay. Continuous measurement is a significant advance because it is more convenient and requires less reagent. Since the optimal pH for RTA to depurinate the naked RNA is pH 4, RNA depurination could only be measured discontinuously [34,36]. However, the assay retains the advantage of greater sensitivity afforded by the use of APRTase and PPDK. Using this method RTA mutants defective in binding ribosomes were identified and RTA residues critical for binding to the ribosomal stalk were mapped [34,36]. The enzymatically coupled assay was also instrumental in demonstrating that the A1 subunit of Stx2 is more active than the A1 subunit of Stx1 on yeast and rat liver ribosomes [31].

## 3. Translation Inhibition Assays

### 3.1. In Vitro Translation Inhibition Assays

Cell free radioactive amino acid incorporation—in vitro translation inhibition was the earliest method to study enzymatic activity of RIPs. Olsnes et al. in 1972 reported that translation inhibition activity of ricin is a downstream result of depurination by measuring incorporation of radioactive amino acids into the newly synthesized protein [66]. The assay was carried out in a rabbit reticulocyte lysate cell free translation system with radioactively labeled amino acids [67]. The newly synthesized proteins were recovered after precipitation with trichloroacetic acid (TCA) and quantified by scintillation counting [68]. Kinetics of rat ribosome inactivation by ricin and abrin was examined using this method [67]. The 60S ribosomal subunit, but not the 40S subunit was found to be the target of ricin [69]. Rat liver ribosomes were shown to be more sensitive to ricin than wheat germ ribosomes, and EF-2 and yeast tRNA were shown to have protective effects against ricin [70].

Bai et al. developed a luciferase synthesis assay to measure the activity of RTA inhibitors in vitro without requiring the use of radioisotopes [71]. This method used capped RNA that encoded luciferase which was transcribed in vitro [71,72]. After incubation of rabbit reticulocyte lysate with the capped RNA encoding luciferase in the presence or absence of RIP, luciferase substrate was used to detect light emission with a luminometer. Bai et al. used it to validate the inhibition by compounds identified in a virtual library screen [73].

### 3.2. Mammalian Cell-Based Assays

#### 3.2.1. In-Cell Radioactive Amino Acid Incorporation Assay

In 2010, Stechmann et al. screened more than 16,000 compounds against ricin and Stx [43] using a conventional cell-based protein synthesis assay. Mammalian cells were exposed to ricin mixed with compounds from the library in the presence of radioactive amino acids and incorporation was quantified by lysing cells, and precipitating the protein followed by scintillation counting [43]. Two compounds, which selectively block the retrograde trafficking of ricin and Stx, were identified [43]. No inhibitors of the toxin enzymatic activity were found.

#### 3.2.2. In-Cell Luciferase Synthesis Assay

In 2005, Zhao et al. developed a nonradioactive assay based on luciferase expression as a measure of protein synthesis [74]. Their assay involved transfection of cell lines with cDNA encoding a destabilized derivative of luciferase with a short half-life. Luciferase cDNA was delivered to cells by adenovirus transduction and the level of protein synthesis was measured using the light output from luciferase [74]. Zhao et al. adapted this assay to 24-, 96-, and 384-well high throughput screening (HTS) format and examined various cell lines for their susceptibility to RIPs and bacterial toxins including Stx, *Pseudomonas* exotoxin A and diphtheria toxin [74]. Saenz et al. used this assay to screen a library of 14,000 small molecules against Stx [44]. Two compounds with inhibitory activity were found to act on intracellular transport steps of Stx [44], demonstrating the feasibility of using nonradioactive reporters to screen for inhibitors of the RIPs.

#### 3.2.3. Cell Based Luciferase Assay

Wahome et al. developed a simplified cell-based luciferase assay in 2010, which did not require transfection of cells with luciferase cDNA [72] prior to seeding. Vero cells were seeded in 384-well plates, incubated overnight and were treated with small molecules followed by ricin. The addition of luciferase plus luciferin substrate resulted in light emission in direct proportion to cellular ATP levels, indicating cell viability. This method was sensitive, had a high signal to background ratio generally ≥10, and was robust and reproducible. Wahome et al. screened more than 200,000 compounds from 17 commercially available chemical libraries against ricin and Stx and found various compounds with wide range of inhibitory effects [72]. However, the majority of compounds could not be confirmed at lower doses in a secondary screen. Several compounds that interfered with ricin in a nonspecific manner, such as by stimulating protein synthesis or by nonspecifically blocking the activity of ricin by sequestering it or by causing partial unfolding, were identified. One inhibitor could block depurination activity in a secondary cell-free translation assay. The majority of the compounds identified interfered with steps in ricin cytotoxicity other than depurination activity, such as cell binding or intracellular trafficking [72].

#### 3.2.4. In-Cell GFP Synthesis Assay

Redmann et al. developed a mammalian cell based assay using green fluorescent protein (GFP) transfection to measure protein synthesis inhibition by ricin [75]. Cells were cotransfected with ricin and a GFP expression plasmid. GFP florescence was quantified using flow cytometry. RTA expression limited the expression of GFP in 70–80% of cells [75]. Jetzt et al. used a slightly modified enhanced green fluorescent protein (EGFP) transfection assay to study ricin mutants in mammalian cells [39,40]. Cells were cotransfected with an EGFP reporter plasmid and RTA mutants and EGFP fluorescent signal was quantified using a plate reader [39,40]. The results correlated well with ribosome depurination measured for the mutants by qRT-PCR. Jetzt et al. found that apoptosis could be triggered by a low level of ribosome depurination [39] and showed that ribosome interactions were critical for RTA toxicity in mammalian cells [40]. The GFP transfection assay [40] was sensitive and useful for examining variations of enzymatic activity among the mutant A1 subunits of Stxs [31]. A stably transfected cell line expressing GFP [76,77] was used for cholera toxin and other type II RIPs in related studies [77,78,79]. However, even with a short-lived mRNA for GFP, the considerable background at the onset of inhibition limited its sensitivity.

## 4. Advantages and Disadvantages of the Different Assays to Measure the Activity of RIPs

The advantages and the disadvantages of the depurination assays are summarized in Table 1. Among the depurination assays, aniline cleavage assay, primer extension assay, and qRT-PCR assay focus on reporting and quantifying the depurinated rRNA template. The aniline cleavage method [16,49] was widely used in early studies. However, it is not very sensitive or quantitative. The primer extension assay [38] was later developed to improve sensitivity, to offer more accurate quantification and to localize the depurination site. However, both assays are laborious and are susceptible to RNase contamination. Compared to the primer extension assay, the qRT-PCR assay does not need radiolabeled primers [55,56]. It stands out for its high sensitivity and wide adaptability for various kinds of samples, though it requires prior knowledge of the depurination site for primer design, it is very useful and relatively easy to establish for RIP studies.

The adenine detection assays, either using HPLC or enzymatically-coupled reactions, focus on detecting the released adenine from the depurination reaction. The HPLC assay [57] is very sensitive but is labor intensive and not suitable for HTS. The HPLC-MS assay [62,63], which does not require adenine derivatization provides a rapid, sensitive, and quantitative assessment of the activity of RIPs and is more amenable to HTS. However, the high cost of HPLC-MS equipment limits its accessibility. The enzymatically coupled assay developed by the Schramm group made the biggest impact in measuring the kinetics of depurination and allowed characterization of kinetic parameters of different RIPs and RIP mutants [65]. However, it required purification of APRTase and PPDK, which are not commercially available. In addition, one of the required reagents, 5-phospho-α-d-ribosyl-1-pyrophosphate (PRPP) is expensive and difficult to obtain. This method is rapid, convenient to perform and amenable to HTS. It cannot be used for in vivo studies but does allow continuous measurement for ribosome depurination as well as rapid determination of RIP enzymatic kinetics. It is adaptable for HTS screening of small molecule inhibitors, but with limitations from interference with the coupling enzymes.

The advantages and the disadvantages of the translation inhibition assays are summarized in Table 2. Since translation inhibition is a downstream effect of depurination, translation inhibition assays do not measure catalytic activity of the RIPs on the ribosome directly. Many early studies used the cell-free radioactive amino acid incorporation assay [66,67,68] to measure translation inhibition by RIPs. Replacing radioactive amino acids using capped RNA encoding luciferase [71] made the assay more convenient and compatible with HTS, but added potential sources of interference by inhibitors, which could interact with luciferase due to structural similarity to the luciferin substrate [80] or inhibit the degradation of luciferase, leading to false positives. These limitations can be eliminated by appropriate controls, but add to the labor of the screen. A highly sensitive detection system independent of luciferase would considerably aid HTS in the future.

To expand the scope of screening for RIP inhibitors cell-based assays were developed to identify compounds affecting mammalian cell sensitivity. Since cell-based assays are affected by the time frame of exposure to the toxins the proper time for toxin treatment or transfection needs to be determined. Many inhibitors identified in cell-based screens act on retrograde trafficking of the toxins because trafficking to the endoplasmic reticulum (ER) is required for ricin and Stxs to enter the cytosol [43,44,72,74,75]. If ATP is used as a measure of cell viability, compounds affecting caspase activity and subsequent steps in apoptosis can interfere either positively or negatively. The in-cell radioactive amino acid incorporation assay [43] does not require transfection of reporter mRNA and is less likely to suffer from nonspecific interference but it is labor intensive because of the requirement for radioactive labeling. The in-cell luciferase [74] and GFP synthesis assays [39,40,75] are sensitive, quantitative, and reproducible and do not require radioactive material. But they require transfection of reporter mRNA, which adds a potentially variable step and has its own time requirement, reducing the likelihood of detecting direct depurination inhibitors. The cell-based luciferase assay [72] does not require transfection and thus is convenient for HTS, but has potential interference by luciferase inhibitors. Avoiding cell transfection reduces labor in HTS format but requires longer elapsed time and leads to higher sample-to-sample variation. Cell-free translation assays have the greatest chance of identifying inhibitors of enzymatic activity of RIPs although translation inhibition step is downstream of depurination.

## 5. Contributions of the Different Assays to Measure Activity to RIP Research

Advances in depurination and translation inhibition assays have broadened the scope of RIP research, and allowed comparison of the activities of RIPs and characterization of the kinetics of depurination. For example, the molecular basis of why Stx2 is more virulent and more frequently associated with HUS than Stx1 was not clearly understood [81,82,83]. The B subunits were shown to be important for the differential toxicity, but the B subunits alone did not account entirely for the differential toxicity [81,82,83]. The potential role of the A1 subunits was not investigated because they did not display differences in translation inhibitory activity in cell-free translation inhibition assays [84,85]. Direct comparison of the enzymatic activity of the A1 subunits was not possible since activation of the holotoxin often yields variable amounts of activated A1 subunits. Furthermore, since translation inhibition is downstream of depurination, translation inhibition assays will not show differences in depurination activity, binding kinetics or affinity of the A1 subunits for the ribosome even though these features can affect toxicity in vivo. Due to technical limitations enzymatic differences between Stx1A1 and Stx2A1 were not observed previously. Using more sensitive techniques, such as qRT-PCR, enzymatically-coupled adenine depurination assay and in-cell EGFP translation inhibition assay, Stx2A1 was shown to have higher catalytic activity than Stx1A1 [31]. Stx2A1 had higher affinity for yeast and rat liver ribosomes and showed higher depurination activity when expressed in yeast and in Vero cells compared to Stx1A1 [31].

Assays that could distinguish between the depurination activity of RIPs on intact ribosomes and on the naked rRNA provided information about the ribosome specificity of RIPs. Ricin depurinates the naked rRNA from all species yet differs widely in its ribosome specificity with the highest activity on mammalian ribosomes [86], lower activity on yeast ribosomes [20], and even lower activity on plant ribosomes [70,87]. Ricin cannot depurinate bacterial ribosomes [88]. These studies suggested that ribosomal proteins determine species specificity of ricin. The contribution of toxin-ribosome interactions to the depurination activity of RIPs was determined by comparing the activity of mutants on ribosomes and an SRL mimic RNA, since depurination activity on rRNA does not require ribosome binding [31,34,35,36,37]. Using the qRT-PCR and the enzymatically-coupled adenine detection assay, mutants defective in ribosome binding, but not catalytic activity were identified and ribosome-binding site of RTA and Stxs were mapped to the distal face of the active site [31,34,35,36,37]. These assays showed that the interaction of Stxs and RTA with the stalk proteins has a profound effect on their catalytic efficiency towards the SRL [34,36,37,89].

Since RIPs like ricin and Stxs are concerns for public health and bioterrorism [19], assays that detect toxin activity are important for diagnosis of STEC infections to prevent food poisoning and for detection and mitigation of bioterrorism threats. It is important for the detection assays to distinguish active and inactive toxins [29]. The assays discussed here are excellent tools to measure bioactivity of the toxins with high sensitivity. Currently two different recombinant RTA vaccines are under development, RiVax and RVEc [90]. One of the major hurdles in the development of these vaccines is assessing vaccine efficacy in humans. Antibody response to ricin is complex since toxin can be neutralizing, non-neutralizing and even enhancing [91]. Epitope specificity, rather than antibody affinity or antibody isotypes, is the primary determinant of neutralization [91]. Toxin-neutralizing activity (TNA), which is a critical measurement of the antibody quality [90], is determined using mammalian cell-based toxicity assays [72,92]. TNAs are reported in different units from different studies, making comparisons difficult [90]. Moreover, TNA levels do not always predict protective immunity. Protection from a lethal dose of ricin challenge can occur in the absence of detectible TNA, suggesting that the limit of detection of cell-based assays may be too low to detect toxin-neutralizing activity [90]. Neutralizing antibodies that interfere with toxin’s enzymatic activity can only be distinguished by assays that directly measure enzymatic activity. Depurination assays using qRT-PCR and the enzymatically-coupled adenine detection assay are two promising choices to improve sensitivity and to identify the exact mechanisms by which ricin is neutralized in vivo.

Considering the large number of assays required for antibody screening, epitope mapping and study of the mechanism of neutralization it is essential to choose a sensitive assay that can directly measure enzymatic activity of the toxin and can be readily performed in a HTS format. Assays adaptable to HTS format are particularly useful for identification of small molecule inhibitors. A handful of small molecule inhibitors of ricin have been discovered using translation inhibition assays [45,72,92] that measure enzymatic activity of the toxin indirectly. So far none of the depurination assays is compatible with HTS and the available translation inhibition HTS methods all have limitations. Improvements that reduce the potential for interference in an HTS method for direct depurination measurement would greatly benefit the search for small molecule inhibitors.

RIPs play a continuing role as unique tools in the development of immunotoxins against cancer [93], the function of ribosomal stalk proteins [42,89,94], ER associated protein degradation (ERAD), retrograde trafficking [8], apoptosis, and the ribotoxic stress response [95]. To employ them as tools to study these processes, it is important to understand the advantages and the disadvantages of variety of assays available to measure their activity. Here we summarize the assays used to measure the depurination and translation inhibition activity of RIPs, explain the advantages and disadvantages of each method and point out how they are used to understand the mechanism of action of RIPs. The information presented here can be used as a guide for selection of methods for screening for RIP therapies and for other basic studies in which RIPs are used as tools.

## Figures and Tables

**Table 1:** Summary of depurination assays.

Method Name	Assay System	RTA Sensitivity (Substrate) *	Detection Method	Advantages	Disadvantages	References
Aniline cleavage	In vitro	Rib: 1 ng rRNA: 10 ng	Polyacrylamide gel separation.	First depurination method. Demonstrated mechanism of action of RIPs.	Labor intensive. Susceptible to RNase contamination. Not adaptable for HTS.	[9,11,16,49]
Primer extension	In vitro In vivo	Rib: 10 ng	Polyacrylamide gel separation. Quantification by PhosphorImager.	More accurate quantification with dual primer. Allows localization of the depurination site.	Labor intensive. Susceptible to RNase contamination. Uses radioactive labeling. Not adaptable to HTS. Requires knowledge of the depurination site for primer design.	[20,38,50,53]
qRT-PCR	In vitro In vivo	Rib: 0.18 ng rRNA: 180 ng	qRT-PCR with either absolute quantification or relative quantification by calculating fold change compared to a control.	Highly sensitive. Accurate quantification. Does not require radioactive material.	Requires knowledge of the site of depurination for primer design.	[34,56]
HPLC	In vitro	SRL: 100ng	Adenine derivatization followed by HPLC or direct adenine detection by HPLC-MS.	Highly sensitive. Allows calculation of enzymatic kinetics. Does not require radioactive material.	Labor intensive and time consuming. Not applicable to in vivo studies. Expensive equipment. Not adaptable to HTS.	[57,61,62]
Enzyme-coupled adenine detection	In vitro	Rib: 1.5 ng SRL: 60 ng	Adenine conversion into readable colorimetric shift signal or luciferase light signal quantified by a microplate reader.	Fast and highly sensitive. Allows continuous measurement of depurination. Does not require radioactive material. Adaptable to HTS.	High background if ingredients not pure. APRTase, PPDK and PRPP can be hard to obtain. Small molecules may interfere with enzymes in inhibitor screens. Saturating ribosome concentrations are hard to obtain. Not applicable to in vivo studies.	[34,36,64,65]

* Rib: purified ribosome. α-sarcin/ricin loop (SRL): An RNA mimic of SRL. Abbreviations: RTA: ricin toxin A chain; RIP: Ribosome-inactivating protein; HTS: high throughput screening; PPDK: Pyruvate orthophosphate dikinase; PRPP: 5-phospho-α-d-ribosyl-1-pyrophosphate.

**Table 2:** Comparison of translation inhibition assays.

Method Name		Advantages	Disadvantages	References
In vitro assays	Radioactive amino acid incorporation	First assay available for RIP activity.	Requires handling of radioactive materials.	[66,67,68,69]
	Luciferase synthesis	Adaptable to HTS. No radioactive materials.	Luciferase can be subject to interference by small inhibitor molecules leading to false positives.	[71,73,80]
Mammalian cell-based assays	In-cell radioactive amino acid incorporation	Adaptable to HTS.	Requires handling of radioactive materials.	[43]
	In-cell luciferase synthesis	Adaptable to HTS. No radioactive materials.	Requires transfection of cells. Low sensitivity, high background and high sample-to-sample variation.	[44,74]
	Cell-based luciferase assay	Adaptable to HTS. No radioactive materials. No cell transfection.	Can yield nonspecific inhibitors.	[72]
	In-cell GFP synthesis	Highly sensitive. Easy detection of GFP.	Requires cell transfection.	[39,40,75]

GFP: green fluorescent protein.
